# Protective Effects of Dihydromyricetin against •OH-Induced Mesenchymal Stem Cells Damage and Mechanistic Chemistry

**DOI:** 10.3390/molecules21050604

**Published:** 2016-05-09

**Authors:** Xican Li, Jingjing Liu, Jian Lin, Tingting Wang, Jieyuan Huang, Yongqiang Lin, Dongfeng Chen

**Affiliations:** 1 School of Chinese Herbal Medicine, Guangzhou University of Chinese Medicine, Guangzhou 510006, China; 15622829151@163.com (J.L.); wtttx0304@163.com (T.W.); jieyuan_huang@163.com (J.H.); yorkoran@foxmail.com (Y.L.); 2 School of Basic Medical Science, Guangzhou University of Chinese Medicine, Guangzhou 510006, China; linjianchn@outlook.com

**Keywords:** dihydromyricetin, mesenchymal stem cells, antioxidant mechanisms, hydroxyl radical-induced, Fe^2+^-chelation, 3–OH group, 2,3-double bond, electron transfer (ET) pathway

## Abstract

As a natural flavonoid in *Ampelopsis grossedentata*, dihydromyricetin (DHM, *2R,3R*-3,5,7,3′,4′,5′-hexahydroxy-2,3-dihydroflavonol) was observed to increase the viability of •OH-treated mesenchymal stem cells using a MTT [3-(4,5-dimethylthiazol-2-yl)-2,5-diphenyl] assay and flow cytometry analysis. This protective effect indicates DHM may be a beneficial agent for cell transplantation therapy. Mechanistic chemistry studies indicated that compared with myricetin, DHM was less effective at ABTS^+^• (2,2′-azino-bis(3-ethylbenzothiazoline-6-sulfonic acid radical) scavenging and reducing Cu^2+^, and had higher •O_2_^−^ and DPPH• (1,1-diphenyl-2-picrylhydrazyl radical) scavenging activities. Additionally, DHM could also chelate Fe^2+^ to give an absorption maximum at 589 nm. Hence, such protective effect of DHM may arise from its antioxidant activities which are thought to occur via direct radical-scavenging and Fe^2+^-chelation. Direct radical-scavenging involves an electron transfer (ET) pathway. The hydrogenation of the 2,3-double bond is hypothesized to reduce the ET process by blocking the formation of a larger π-π conjugative system. The glycosidation of the 3–OH in myricitrin is assumed to sterically hinder atom transfer in the •O_2_^−^ and DPPH• radical-scavenging processes. In DHM, the Fe^2+^-chelating effect can actually be attributed to the 5,3′,4′,5′–OH and 4–C=O groups, and the 3–OH group itself can neither scavenge radicals nor chelate metal.

## 1. Introduction

It is well documented that bone marrow-derived mesenchymal stem cell (bmMSC) transplantation represents one of the more promising new strategies in the treatment of various diseases such as neuronal regeneration [1,2], brain damage [3,4], liver damage [5], and heart failure [6]. In the process of transplantation, various reactive oxygen species (ROS) may cause oxidative damage and apoptosis [7]. These ROS mainly include superoxide radical anion (•O_2_^−^), peroxynitrite (ONOO^−^), hydroxyl radical (•OH), and so on. 

The •O_2_^−^ and ONOO^−^ radicals however have recently been reported to be inhibited by the antioxidant defense system in bmMSCs to some extent [2,4]. Thus, bmMSCs themselves had antioxidant potential to decrease oxidative damage and apoptotic death [5]. However, such endogenous antioxidant potential cannot guarantee the cell survival for their clinical applications in cell transplantation and tissue engineering. Other more toxic ROS, especially hydroxyl radical (•OH), can severely damage the cells and result in poor cell survival following transplantation. The resistance to •OH-induced cellular damage or apoptosis may mainly rely on some exogenous effective antioxidants, such as natural flavonoids.

**Figure 1:**
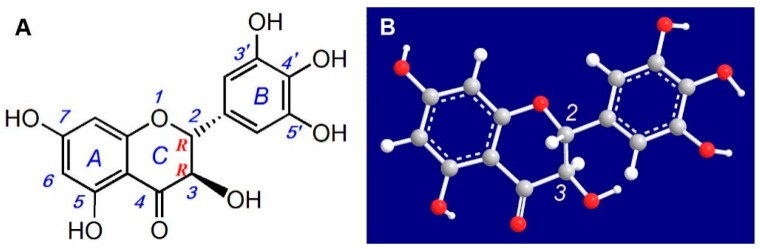
The chemical structure (**A**) and ball-stick model (**B**) of dihydromyricetin (DHM, *2R*,*3R*-3,5,7,3’,4’,5’-hexahydroxy-2,3-dihydroflavonol).

As a main flavonoid, dihydromyricetin (DHM, Figure 1A) occurs in *Ampelopsis grossedentata* (Hand-Mazz.) [8], and has attracted increasing attention owing to its beneficial effect on cells. It has been recently reported that DHM can protect endothelial cells from H_2_O_2_-induced oxidative damage via mitochondrial pathways [9], and exert antioxidant action in soybean oil and cooked ground beef model systems [10]. Additionally, the parent plant *A. grossedentata* has been consumed as a tea (Rattan Tea, Vine Tea, Tengcha) with health benefits in South China [11,12]. It is documented to be able to clear *heat* and remove *toxic* in the body according to Tradition Chinese Medicine (TCM) [11]. The so-called *heat* and *toxic* properties in TCM have been demonstrated by free radical biology to result from oxidative stress induced by ROS, especially the •OH radical [13]. Taken together, these observations suggest that DHM may possess a beneficial effect on the apoptosis of MSCs, although to our knowledge, there is no direct evidence to support this. Therefore, we investigated the beneficial effects of DHM on the viability of •OH-treated MSCs using the MTT [3-(4,5-dimethylthiazol-2-yl)-2,5-diphenyl] assay and flow cytometry analysis.

It must be emphasized that the structure of DHM has not been reported consistently in the literature. Zhu reported a structure with a double bond between the 2- and 3-positions of the DHM molecule [14]. Shen and colleagues incorrectly reported the stereo configuration of DHM [15]. Several other reports mentioned no stereo configuration for DHM [16,17,18]. These structural inconsistencies and errors may lead to misunderstandings about some of the chemical moieties in the DHM molecule. For example, the structure drawn by Zhu is actually myricetin, not dihydromyricetin (DHM) and the 3–OH was wrongly identified as a phenolic –OH group [14]. Zhang even stated that the 3–OH along with other –OH groups and a –C=O played a critical role in antioxidant action of DHM [8]. Therefore, the reliability of these results has been questioned. 

Herein, we use a ball-stick model to intuitively show the stereo configuration of DHM. As illustrated in Figure 1B, the absolute configurations of C-2 and C-3 are (*R*,*R*), and the full IUPAC name of DHM is (*2R*,*3R*)-3,5,7,3’,4’,5’-hexahydroxy-2,3-dihydroflavonol. Our detailed drawing should be helpful to understand the role of the 3–OH in DHM and other dihydroflavonoids. Furthermore, this work will support the screening of natural dihydroflavonoids and their synthetic derivatives as effective antioxidants for cell transplantation and tissue engineering purposes.

## 2. Results and Discussion

Chemical or physical environments such as radiation and iron overload can damage MSCs during the process of proliferation and differentiation because of the generation of the •OH radical [19,20,21]. For example, radiotherapy for cancer patients has been indicated to decrease cell survival in autologous stem cell transplantation [22]; Iron overload can yield the •OH radical through the Fenton reaction (Equation (1)) or Haber-Weiss (Equation (2)). In the present study, Fenton’s reagent (FeCl_2_ *plus* H_2_O_2_) was used to damage MSCs, and then the MTT assay was firstly used to evaluate the beneficial effect of DHM on •OH radical-treated MSCs:

Fe^2+^ + H_2_O_2_ → Fe^3+^ + OH^−^ + •OH (1)

•O_2_^−^ + H_2_O_2_ → OH^−^ + •OH + O_2_ (2)

**Figure 2:**
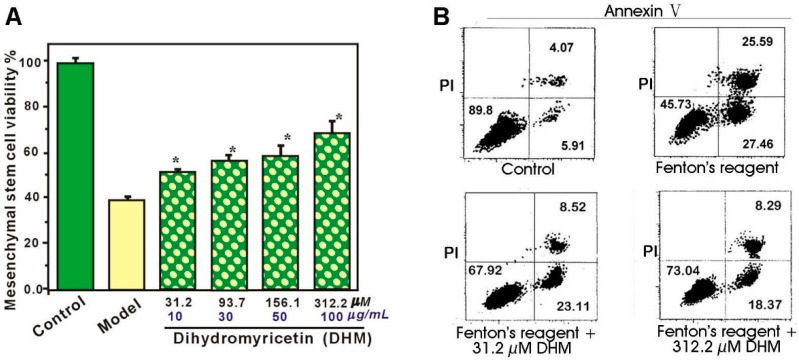
Dihydromyricetin (DHM) protects MSCs from Fenton’s reagent-induced damage (or apoptosis): (**A**) MTT assay. Fenton’s reagent refers to FeCl_2_ *plus* H_2_O_2_. Experiments were performed with three batches of cells and each batch was tested in triplicate. Data are the mean ± SD. * *p* < 0.05, compared with MSC damage following Fenton’s reagent; (**B**) Flow cytometry analysis. Fenton’s reagent refers to FeCl_2_ *plus* H_2_O_2_. MSCs were treated with or without Fenton’s reagent in the absence or presence of DHM at the indicated concentrations. The apoptotic cells were analyzed by means of flow cytometry for annexin V and PI. This assay was performed to distinguish intact cells (annexin V−/PI−), necrotic (annexin V−/PI+) cells, early apoptotic (annexin V+/PI−) cells, and late apoptotic/necrotic (annexin V+/PI+) cells. Experiments were performed with two batches of cells and each batch was tested in duplicate. Data are shown as the mean ± SD (*n* = 4). * *p* < 0.05, compared with the Fenton’s reagent treatment group.

As shown in Figure 2A, the viability of MSCs in the control group was 100%. By comparison, the model group treated with Fenton’s reagent exhibited the lowest cell viability (40.3%) indicating that the MSCs in the model group were oxidatively damaged. However, the viabilities of MSCs in the sample groups (31.2–312.2 µM DHM) were effectively restored indicating an anti-apoptotic effect of DHM on MSCs. The anti-apoptotic effect was further investigated using flow cytometry. As shown in Figure 2B, MSC apoptosis was quantified by staining the cells with annexin-V-FITC/PI. The number of annexin V-positive cells was 9.0% ± 1.5% in MSCs treated without FeCl_2_ and H_2_O_2_, 53% ± 2.6% in MSCs treated with FeCl_2_ and H_2_O_2_, and 32% ± 2.7% and 26% ± 3.1% in MSCs treated with H_2_O_2_ and FeCl_2_ in the presence of the indicated concentrations of DHM. There was a significant difference among the groups, post hoc comparisons revealed that the apoptotic cell number of the MSCs treated with H_2_O_2_ and FeCl_2_ was increased and this increase could be inhibited by DHM (*p* < 0.05).

These observations partly support why DHM protected PC12 cells from apoptosis induced by SNP, a reagent which can release massive amounts of the free radical NO [23]. This further supports the fact that Tengcha tea can prevent hypolipidemia in mice [12], and FeSO_4_-edetic acid-induced lipid peroxidation in the presence of linoleic acid [8]. More importantly, these results support the potential application of DHM in MSC transplantation therapy, especially when the cells have been subjected to radiation (especially in radiotherapy), iron overload, or other oxidative stress factors.

**Figure 3:**
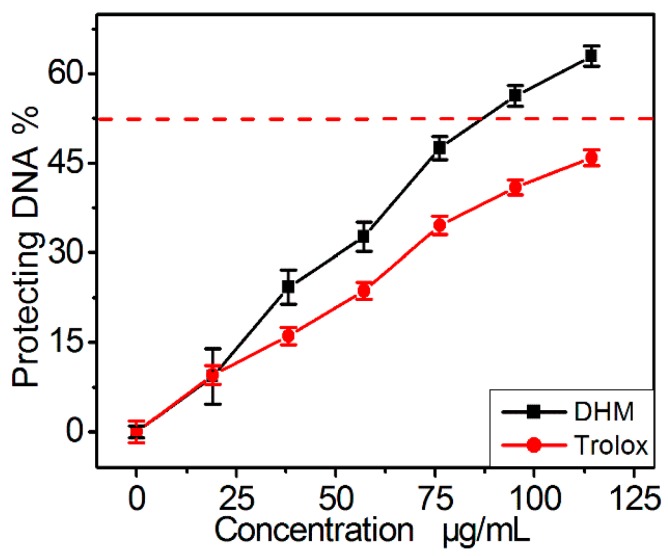
The protective effect of dihydromyricetin (DHM) against •OH-induced DNA damage *in vitro.* Each value is expressed as the mean ± SD (*n* = 3).

Additionally, such protection has been demonstrated to be partly attributed to the repair of DNA oxidative damage [24]. As shown in Figure 3, DHM exhibited a protective effect against DNA damage in a concentration-dependent manner *in vitro*. Its protective effect on DNA is reported to be directly associated with its fast repairing DNA radical and ROS scavenging (antioxidant) effect [25], and may be partly responsible for its anticancer effect because cancer and aging are closely related to •OH radical-induced damage to cells and DNA [14,26,27].

In the aspect of antioxidant effect, the value of the ratio of IC_50,Trolox_/IC_50,DHM_ (Table 1) revealed that DHM possessed 1.8-fold higher relative antioxidant effect the standard antioxidant Trolox. This is similar to its analogue myricitrin (Figure S1) which exhibited 1.7-fold higher antioxidant activity compared with Trolox (Table 1) [26]. Although myricitrin has a glucoside at C-3 and a double bond between the 2- and 3-positions, its antioxidant activities were similar to those of DHM. Thus, the antioxidant activities may result from the extreme reactivity of the •OH radical, which can quickly and completely damage any nearby molecules or molecular moieties. Therefore, chemical moieties such as a double bond, sugar residues (glucosides), or *R*/*S* configuration would not give distinguishing antioxidant results.

Because catalytic iron (especially Fe^2+^) can accelerate the generation of •OH radicals in the Fenton reaction or Haber-Weiss reaction (Equations (1) and (2)), Fe^2+^-chelation can decrease the level of •OH radicals, and is regarded as indirect means of scavenging •OH radicals [28]. 

**Figure 4:**
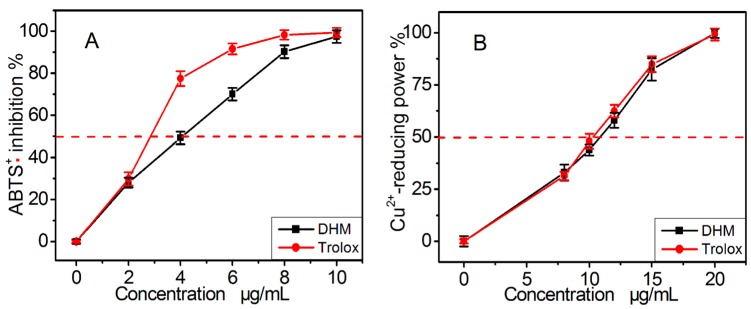
The dose response curves of dihydromyricetin (DHM) and positive control Trolox in the ABTS•^+^ radical-scavenging assay (**A**) and Cu^2+^-reducing power assay (**B**). Each value is expressed as the mean ± SD (*n* = 3). Trolox, [(±)-6-hydroxyl-2,5,7,8-tetramethlychroman-2-carboxylic acid].

To test the possibility that DHM may act as a direct radical scavenger, we used the ABTS^+^• radical-scavenging assay where the ABTS^+^• radical can be generated without a metal catalyst. As shown in Figure 4A, DHM inhibited ABTS^+^• radical scavenging in a dose-dependent manner from 2 to 10 μg/mL. However, the IC_50_ ratio (0.5 *vs* 1.8) showed that its ability was weaker than that of myricitrin. This may be linked to differences in the mechanism of ABTS^+^• scavenging between the two compounds.

**Figure 5:**
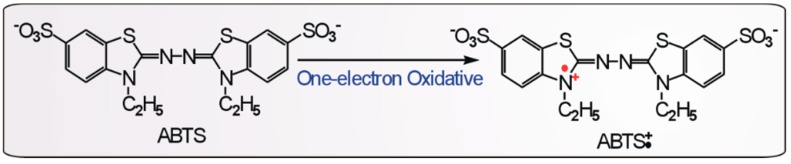
The one-electron transfer from ABTS to ABTS^+^• [30].

**Figure 6:**
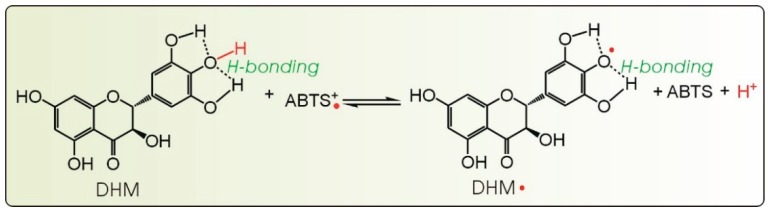
The proposed reaction of DHM with the ABTS^+^• radical cation (initial stage).

ABTS^+^• radical cation scavenging is considered to be an electron transfer (ET) process [29], similar to its formation, which was previously proven to be via one-electron oxidation from ABTS [30] (Figure 5). Based on this knowledge and the ABTS^+^• reaction with the pyrogallol moiety [31,32], we proposed the initial stage of the DHM reaction with the ABTS^+^• radical cation to occur as shown in Figure 6.

To further test the possibility of an ET process occurring during radical scavenging, DHM was investigated using a Cu^2+^-reducing assay. The metal reducing reaction is known as an ET process, and the Cu^2+^-reducing assay is normally utilized to explore the possibility of whether an antioxidant can act via an ET process [33]. The observations that DHM could effectively reduce Cu^2+^ to Cu^+^ at concentrations of 8–20 μg/mL (Figure 4B), further verified the possibility it acts via ET. Of course, the ET may also have a role in the repairing DNA radical by DHM [25].

However, the possibility that DHM participates in an ET process was less likely than that of myricitrin, as suggested by the IC_50_ ratios in Table 1 (0.5 *vs* 1.8 and 1.3 *vs* 3.0, for the ABTS^+^• and Cu^2+^-reducing assay, respectively). After ET, DHM would be transformed into a DHM• radical. The DHM• radical is actually a phenoxy• radical which needs a large conjugative system for delocalization and stabilization. However, as shown in Figure 1B, the 2,3-double bond saturated with two hydrogen atoms might block the formation of a larger conjugative system with *C*&*A* rings. Hence, the DHM• radical would be unstable, and this instability would partially prevent the occurrence of the ET pathway. Myricitrin, containing a 2,3-double bond (Figure S1), can form a larger conjugative system with *C* & *A* rings to delocalize and stabilize the phenoxy• radical. Therefore, myricitrin is more likely to undergo ET, and have stronger ABTS^+^• scavenging and Cu^2+^-reducing abilities regardless of the presence of a large sterically hindered glucoside.

In addition to the ET pathway, an atom transfer pathway has also been reported to play a role in the antioxidant action of phenolics [34]. Atom transfer can be further classified into several subtypes: hydrogen atom transfer (HAT), concerted proton-coupled electron transfer (CPCET), electron transfer followed by proton transfer (ET-PT), proton transfer followed by electron transfer (PT-ET), and sequential proton loss electron transfer (SPLET) among others [35]. As a stable free radical, DPPH• can be reduced by phytophenols to DPPH-H (Equation (3)) [36]. This is demonstrated to be an atom transfer [37], and thus, we analyzed the DPPH• scavenging ability of DHM:

DPPH• + phytophenol-OH → DPPH-H + phytophenol-O•
(3)

**Figure 7:**
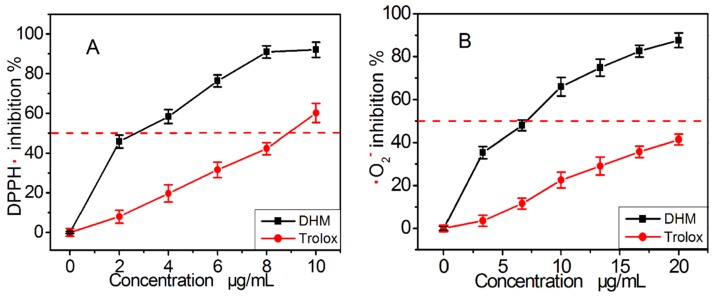
The dose response curves of dihydromyricetin (DHM) and the positive control Trolox in the DPPH• scavenging assay (**A**) and •O_2_^−^ scavenging assay (**B**). Each value is expressed as the mean ± SD (*n* = 3). Trolox, [(±)-6-hydroxyl-2,5,7,8-tetramethlychroman-2-carboxylic acid].

As shown in Figure 7A, DHM scavenged DPPH• radical in a concentration dependent manner from 2 to 10 μg/mL. This indicates that DHM acted through the atom transfer pathway to exert its antioxidant action. The IC_50_ value ratios in Table 1 suggested that DHM was more effective (4.7 *vs* 1.2) than myricitrin. Thus, relative to myricitrin, DHM might be more prone to act via the atom transfer pathway in the DPPH•-scavenging process. We presumed that the C-3 glucoside in myricitrin might sterically hinder the atom transfer. This presumption is further confirmed by our observation in the •O_2_^−^ scavenging assay (Figure 7B) where DHM possessed a stronger (4.7 *vs* 1.2) •O_2_^−^ scavenging effect than myricitrin (Table 1). The atom transfer pathway has been proven to play a role in •O_2_^−^ scavenging [38]. Our data are in accordance with previous findings that indicated that the glycosidation of the 3–OH will promote its stability during extraction [39].

Hydrogenation of the 2,3-double bond in a flavonoid diminishes its ability to participate in ET while glycosidation of the 3–OH can reduce its ability to participate in an atom transfer. Thus, the viewpoint that the 2,3-double bond does not play an important role in the improvement of a flavonoid’s antioxidant ability is inappropriate [40]. Instead, in an ET-favored antioxidant process, the 2,3-double bond plays an important role while in an atom transfer-favored antioxidant process, the 2,3-double bond plays a minor role.

**Figure 8:**
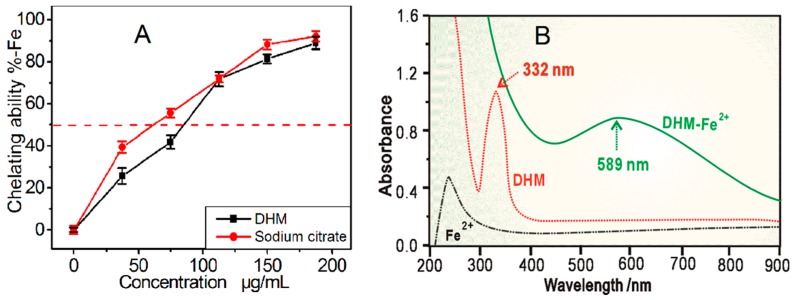
(**A**) The dose response curve of DHM in the Fe^2+^-chelating assay. Sodium citrate is used as the positive control. Each value in (**A**) is expressed as the mean ± SD (*n* = 3); (**B**) UV spectra of dihydromyricetin (DHM) and the DHM-Fe^2+^ complex.

**Figure 9:**
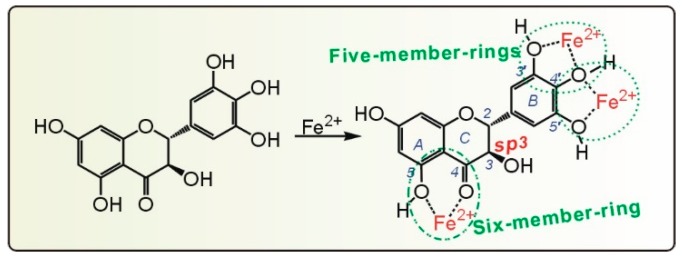
The proposed chelation of Fe^2+^ by dihydromyricetin (DHM).

As mentioned above, metal chelation is one approach for phytophenols to scavenge ROS in cells. Therefore, we studied DHM using a Fe^2+^-chelating assay. As shown in Figure 8A, DHM chelated Fe^2+^ in a dose-dependent manner from 37.5 to 187.5 μg/mL. Compared with sodium citrate (a very strong chelator), DHM has 0.8-times the relative chelating level (Table 1). This indicates that DHM is a reasonable Fe^2+^-chelator and Fe^2+^-chelation may be another mechanism for DHM to scavenge ROS. In our experiment, the DHM-Fe^2+^ complex with its bluish purple color (Figure S2) had an absorption maximum at 589 nm, while DHM with its very light yellow color (Figure S3) had an absorption maximum at 332 nm (Figure 8B). The large bathochromic shift (λ_max_ 332→589 nm) obviously indicated an extension of aromatic conjugation. Based on our data and previous reports [41,42], the Fe^2+^-chelating activity of DHM was proposed to arise from *ortho*- or adjacent hydroxyl groups and a carbonyl group, *i.e.*, the 5,3′,4′,5′–OH groups and the 4–C=O group (Figure 9). These *sp*^2^ carbons have a planar configuration and can readily form a stable five- or six-member ring through the chelation of Fe^2+^. However, the C-3 is *sp*^3^ hybridized, making it an *R-*configuration (not a planar configuration) (Figure 1B). Therefore, the 3–OH group does not share a plan with the adjacent carbonyl group (4–C=O), making it difficult to form a stable ring-shaped Fe^2+^-complex with the 4–C=O. Our interpretation is further supported by the previous finding that the 2,3-double bond was essential for stable copper chelation [43].

Additionally, the 3–OH group is unlikely to undergo hemolytic rupture to produce a hydrogen radical (•H) and alkoxyl radical (•OR), because it is an alcoholic –OH group not a phenolic –OH. Thus, the view that the 3–OH group along with the 5,3′,4′,5′–OH and 4–C=O groups plays an important role in the antioxidant of DHM is not reasonable [8]. 

## 3. Materials and Methods

### 3.1. Chemicals and Animals

Dihydromyricetin (DHM, CAS 27200-12-0, 98%) was purchased from Chengdu Biopurify Phytochemicals Ltd. (Chengdu, China). Dulbecco’s modified Eagle’s medium (DMEM) and fetal bovine serum (FBS) were purchased from Gibco (Grand Island, NY, USA). CD44 was from Wuhan Boster Co., Ltd. (Wuhan, China). Annexin V/propidium iodide (PI) assay kit was purchased from Invitrogen (Carlsbad, CA, USA). DPPH• (1,1-diphenyl-2-picrylhydrazyl radical), neocuproine (2,9-dimethyl-1,10-phenanthroline hemihydrate), Trolox [(±)-6-hydroxyl-2,5,7,8-tetramethlychroman-2-carboxylic acid], ferrozine [3-(2-pyridyl)-5,6-bis(4-phenylsulfonicacid)-1,2,4-triazine], 3-(4,5-dimethylthiazol-2-yl)-2,5-diphenyl (MTT), and pyrogallol were purchased from Sigma Co. (Sigma-Aldrich Shanghai Trading Co., Shanghai, China); 2,2′-Azino-bis(3-ethylbenzothiazoline-6-sulfonic acid diammonium salt) (ABTS) was obtained from Amresco Inc. (Solon, OH, USA); DNA sodium salt (fish sperm) was purchased from Aladdin Chemistry Co. (Shanghai, China); hydrogen peroxide (H_2_O_2_, AR), ferrous chloride (FeCl_2_∙4H_2_O, AR), and dimethyl sulfoxide (DMSO, AR) were from Guangzhou Chemical Reagent Factory (Guangzhou, China). Four-week-old Sprague-Dawley (SD) rats were obtained from the animal center of Guangzhou University of Chinese Medicine. Procurement, maintenance and treatments to animals were performed under the supervision of the Institutional Animal Ethics Committee in Guangzhou University of Chinese Medicine.

### 3.2. Protective Effect Against •OH-Induced Damage to Mscs (MTT Assay)

MSC culture and the MTT assay were carried out according to our previous report [44]. In brief, bone marrow was obtained from the femur and tibia of rat. The marrow samples were diluted with DMEM (LG: low glucose) containing 10% FBS. MSCs were prepared by gradient centrifugation at 900× *g* for 30 min on Percoll of a density of 1.073 g/mL. The cells were washed, counted, and plated at 1 × 10^6^/cm^2^ on Petri dishes in DMEM-LG supplemented with 10% FBS. The medium was replaced and the unattached cells were removed every 3 days. MSCs formed as confluent layers were detached by treatment with 0.25% trypsin and passaged into culture flasks at 1 × 10^4^/cm^2^. MSCs at passage 3 were evaluated for cultured cell homogeneity using detection of CD44 by flow cytometry and used for the MTT assay. For the assay, the cultured MSCs were divided into control, model, and sample DHM groups. In the control group, MSCs were incubated for 24 h in DMEM. In the model and sample groups, MSCs were treated with FeCl_2_ (100 μM) followed by H_2_O_2_ (50 μM). After incubation for 25 min, the mixture of FeCl_2_ *plus* H_2_O_2_ was removed. MSCs in the model group were incubated for 24 h in DMEM, while MSCs in the sample group were incubated for 24 h in DMEM with various DHM concentrations. After incubation, 20 μL MTT (5 mg/mL in PBS) was added and the culture was incubated for a further 3 h. The culture medium was discarded and replaced with 150 μL DMSO. Absorbance at 490 nm was measured using a Bio-Kinetics reader (PE-1420; Bio-Kinetics Corporation, Sioux Center, IA, USA). The culture with the serum medium was used for the control group and each sample test was repeated in five independent wells. The cell viability (% control) was calculated based on the A_490nm_.

### 3.3. Flow Cytometry Analysis for Annexin V and PI

In the control group, MSCs were incubated in DMEM. In the model and sample groups, MSCs were treated with FeCl_2_ (100 μM) followed by H_2_O_2_ (50 μM). After incubation for 25 min, the mixture of FeCl_2_ and H_2_O_2_ was removed. MSCs in the model group were incubated for 24 h in DMEM, while MSCs in the sample group were incubated for 24 h in DMEM with various DHM concentrations. After culture, cells were harvested by trypsin (0.05%) digestion in phosphate-buffered saline (PBS). The cells were washed and re-suspended in PBS, fixed in 70% ethanol and washed with PBS. In the next step, they were labeled with Annexin V and 50 μg/mL PI and ribonuclease (RNase) (10 μg/mL) and then incubated at 37 °C for 45 min. Fluorescence was measured using flow cytometry (EPICS XL, Coulter, Pasadena, CA, USA) with standard software. Experiments were performed with 2 different batches of cells and each batch was tested in duplicate.

### 3.4. Protective Effect against Hydroxyl-Induced DNA Damage (DNA Protection Assay)

The protective effect against •OH-induced DNA damage was estimated using the method developed by our laboratory [45]. Briefly, methanol sample solutions (0.1 mg/mL, 20–120 μL) were separately aliquoted into mini tubes. After completely evaporating the methanol solvent in each tube at 60 °C, the sample residue was treated with 300 μL phosphate buffer (0.2 M, pH 7.4), followed by 100 μL DNA sodium (10 mg/mL), 75 μL H_2_O_2_ (33.6 mM), 50 μL FeCl_3_ (3.2 mM), 100 μL Na_2_EDTA (0.5 mM), and 75 μL ascorbic acid (12 mM). The total volume of the reaction mixture was brought up to 800 μL with buffer. After incubation at 55 °C for 20 min, 250 μL of trichloroacetic acid (10%, *w*/*w*) was added to the tube. After heating the mixture at 105 °C for 15 min with 150 μL 2-thiobarbituric acid (TBA, 5% in 1.25% NaOH aqueous solution), the absorbance was measured at 530 nm (Unico 2100, Shanghai, China) against the buffer (as the blank). The protective percentage is expressed as follows:

(4) $$\mathrm{Protective}\mathrm{effect}\%=\frac{A_{0}-A}{A_{0}}\times 100\%$$

where A_0_ indicates the absorbance of the blank and A indicates the absorbance of the sample.

### 3.5. Pyrogallol Autoxidation Assay for •O^2−^ Scavenging

The pyrogallol autoxidation assay is used to evaluate the superoxide anion (•O_2_^−^) scavenging activity of antioxidants and was previously established by our laboratory [46]. Briefly, 0–600 μL of sample solution (0.1 mg/mL) was prepared in Tris-HCl buffer (0.05 M, pH 7.4) containing Na_2_EDTA (1 mM) in a total volume of 2950 μL. Then about 50 μL pyrogallol solution (60 mM, in 1 mM HCl) was added to the 2950 μL mixture. After vigorous mixing, the absorbance was read at 325 nm every 30 s for 5 min. The •O_2_^−^ scavenging ability was calculated as:

(5) $$\mathrm{Inhibition}\%=\frac{\Delta A_{325\text{nm},\text{control}}-\Delta A_{325,\text{sample}}}{\Delta A_{325,\text{control}}}\times 100\%$$

where, ΔA_325nm,control_ is the increase in A_325nm_ of the mixture without the sample and ΔA_325nm,sample_ is that with the sample.

### 3.6. Cu^2+^-Reducing Power Assay

The Cu^2+^-reducing power assay was carried out according to a previously published method [47]. To 125 μL of CuSO_4_ aqueous solution (10 mM), 125 μL neocuproine solution (7.5 mM in CH_3_OH) and sample solutions (0.1 mg/mL, 0–200 μL) at appropriate concentrations were added. Then, the total volume was adjusted to 1000 μL with the buffer and mixed vigorously. Absorbance against a buffer blank was measured at 450 nm after 30 min. The relative Cu^2+^-reducing power was calculated using the formula:

(6) $$\mathrm{Relative}\mathrm{reducing}\mathrm{power}\%=\frac{A-A_{\text{min}}}{A_{\text{max}}-A_{\text{min}}}\times 100\%$$

where, A_max_ is the maximum absorbance and A_min_ is the minimum absorbance in the test. A is the absorbance of sample.

### 3.7. DPPH• Radical-Scavenging Assay

The scavenging activity on DPPH• free radicals was assessed according to the method reported by Li [48]. Briefly, 50 μL of the methanolic sample solution (at least 5 different concentrations were prepared, 30–150 μL) was mixed with 100 μL DPPH• solution (100 μM in methanol, prepared daily). The mixture was shaken vigorously and left to stand for 30 min in the dark, and the absorbance was then measured at 519 nm against a blank. The DPPH•-scavenging activity of each solution was calculated as the percent inhibition according to the following equation:

(7) $$\mathrm{Protective}\mathrm{effect}\%=\frac{A_{0}-A}{A_{0}}\times 100\%$$

where A_0_ and A are the absorbance of the system in the absence and presence of sample, respectively.

### 3.8. ABTS^+^• Radical-Scavenging Assay

The ABTS^+^• scavenging activity was evaluated according to a previously published method [49]. The ABTS^+^• was produced by mixing 200 μL ABTS diammonium salt (7.4 mM) with 200 μL K_2_S_2_O_8_ (2.6 mM). After incubation in the dark for 12 h, the mixture was diluted with methanol (about 1:50) so that its absorbance at 734 nm was 0.70 ± 0.02. Then, the diluted ABTS^+^• solution (800 μL) was added to 200 μL of an ethanolic sample solution at various concentrations, and thoroughly mixed. After the reaction mixture stood for 6 min, the absorbance at 734 nm was read on a spectrophotometer. The percentage inhibition was calculated by the same formula used for the DPPH• radical-scavenging assay.

### 3.9. Colorimetry Determination and UV Spectra Determination of Fe^2+^-Chelation

The Fe^2+^ chelating activity of DHM was estimated by a colorimetric method [50]. Briefly, 0–150 μL of sample solution (0.1 mg/mL in methanol) was added to 100 μL FeCl_2_ aqueous solution (250 μM). The reaction was initiated by the addition of 150 μL ferrozine aqueous solution (1 mM) and the total volume of the system was adjusted to 1000 μL with methanol. Then, the mixture was shaken vigorously and incubated at room temperature for 10 min. The absorbance of the solution was recorded at 562 nm (Unico 2100). The percentage of the chelating effect was calculated by the formula described in the *DNA protection assay* section. UV spectra analysis was also completed on the DHM-Fe^2+^ complex. A total of 200 μL methanol DHM solution (1 mg/mL) was added to 300 μL FeCl_2_•4H_2_O aqueous solution. Then the mixture was ultrasonicated then centrifuged at 6500× *g* for 10 min. The supernatant spectrum was obtained using a UV/Vis spectrophotometer (Jinhua 754 PC, Shanghai, China). 

### 3.10. Statistical Analysis

Data are given as the mean ± SD of three measurements. The IC_50_ values were calculated by linear regression analysis using Origin 6.0 professional software (Northampton, MA, USA). Determination of significant differences between the mean IC_50_ values of the sample and the positive control was performed using one-way ANOVA. The analysis was performed using SPSS software 13.0 (SPSS Inc., Chicago, IL, USA) for Windows. A value of *p* < 0.05 was considered to be statistically significant.

## 4. Conclusions

DHM can effectively protect MSCs against •OH-induced damage. This protective effect suggests a potential role for DHM in MSC transplantation therapy, and may be attributed to its antioxidant ability. The possible antioxidant mechanisms are thought to include direct radical-scavenging and Fe^2+^-chelation. Direct radical-scavenging uses an ET pathway. The hydrogenation of the 2,3-double bond is hypothesized to reduce the ET by blocking the formation of a larger π-π conjugative system. The glycosidation of the 3–OH in myricitrin is assumed to sterically hinder atom transfer in •O_2_^−^ and DPPH• radical scavenging. Therefore, hydrogenation of the flavonoid 2,3-double bond diminishes its ability to scavenge radicals via ET, while glycosidation of the 3–OH can reduce its ability to participate in atom transfer. In DHM, the Fe^2+^-chelating effect can actually be attributed to the 5,3’,4’,5’–OH and 4–C=O groups, and the 3–OH group itself can neither scavenge radicals nor chelate metal. 

## Figures and Tables

**Table 1:** The IC_50_ values of dihydromyricetin (DHM) and Trolox in various assays.

Assays	DHM	Positive Control	IC_50,Trolox_/IC_50,DHM_	IC_50,Trolox_/IC_50, myricitrin_ *
	μg/mL (μM)	Trolox, µg/mL (μM)		
DNA assay	85.7 ± 2.2 (266.7 ± 0.5 ^a^)	120.6 ± 0.7(483.3 ± 0.5 ^b^)	1.8	1.7
ABTS^+^ scavenging	4.1 ± 0.4 (12.8 ± 0.1 ^b^)	1.7 ± 0.1 (6.8 ± 0.1 ^a^)	0.5	1.8
Cu^2+^-reducing	10.7 ± 0.1(33.4 ± 2.44 ^a^)	10.3 ± 0.1(41.2 ± 0.9 ^b^)	1.3	3.0
·O_2_^−^ scavenging	6.7 ± 0.2 (20.0 ± 0.4 ^a^)	23.2 ± 2.6(90.1 ± 0.5 ^b^)	4.5	1.9
DPPH· scavenging	2.3 ± 0.8 (7.4 ± 0.3 ^a^)	8.78 ± 0.2 (35.1 ± 0.4 ^b^)	4.7	1.2
Fe^2+^ chelating	85.7 ± 1.6(266.7 ± 0.01 ^b^)	59.1 ± 1.3 (200.1 ± 0.5 ^a^) **	0.8	No detected

Each IC_50_ value is expressed as the mean ± SD (*n* = 3). * The IC_50_ values of myricitrin are from [25], and converted from µg/mL to μM. Mean values in brackets with different superscripts (a or b) in the same row are significantly different (*p* < 0.05), while those with same superscripts are not significantly different (*p* < 0.05). ** Sodium citrate, not Trolox.

## Supplementary Materials

Supplementary materials can be accessed at: http://www.mdpi.com/1420-3049/21/5/604/s1.

## Abbreviations

The following abbreviations are used in this manuscript:

ABTS: 2,2′-azino-bis (3-ethylbenzothiazoline-6-sulfonic acid
BHA: butylated hydroxyanisole
bmMSCs: bone marrow derived mesenchymal stem cells
DHM: Dhydromyricetin
DMEM: Dulbecco’s modified Eagle’s medium
DPPH: 1,1-diphenyl-2-picrylhydrazyl radical
ET: electron transfer
FBS: fetal bovine serum
Ferrozine: 3-(2-pyridyl)-5, 6-bis (4-phenylsulfonicacid)-1,2,4-triazine
MSCs: mesenchymal stem cells
MTT: [3-(4,5-dimethylthiazol-2-yl)-2,5-diphenyl]
ROS: reactive oxygen species
SD: standard deviation
TBA: 2-thiobarbituric acid
TCM: Tradition Chinese Medicine
Tris: Tri-hydroxymethyl amino methane
Trolox: (±)-6-hydroxyl-2,5,7,8-tetramethlychroman-2-carboxylic acid
