# Supplementary material for: Protective Effects of Dihydromyricetin against •OH-Induced Mesenchymal Stem Cells Damage and Mechanistic Chemistry
Source: Molecules. 2016 May 9;21(5):604. doi: 10.3390/molecules21050604 (PMC6274564; doi:10.3390/molecules21050604)
Supplement: Supplementary file 1 [file molecules-21-00604-s001.pdf]

Supplementary Materials: Protective Effects of Dihydromyricetin against •OH-Induced Mesenchymal Stem Cells Damage and Mechanistic Chemistry

Xican Li, Jingjing Liu, Jian Lin, Tingting Wang, Jieyuan Huang, Yongqiang Lin and Dongfeng Chen

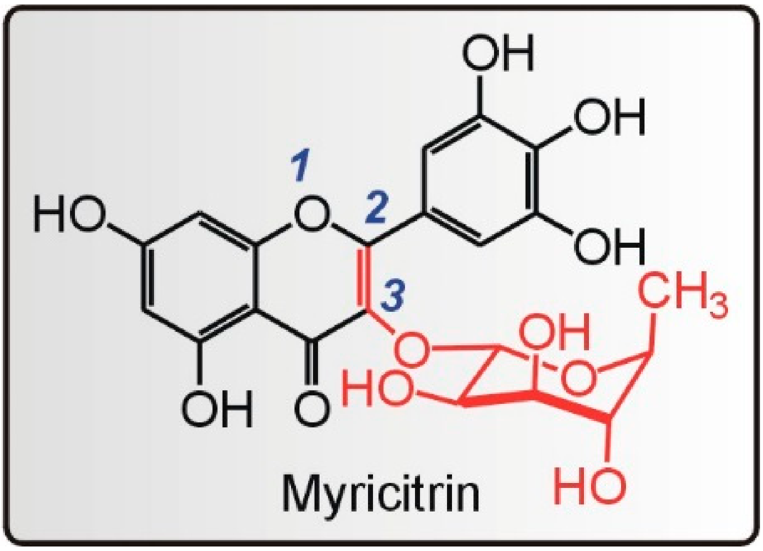

Figure S1. The chemical structure of myricitrin.

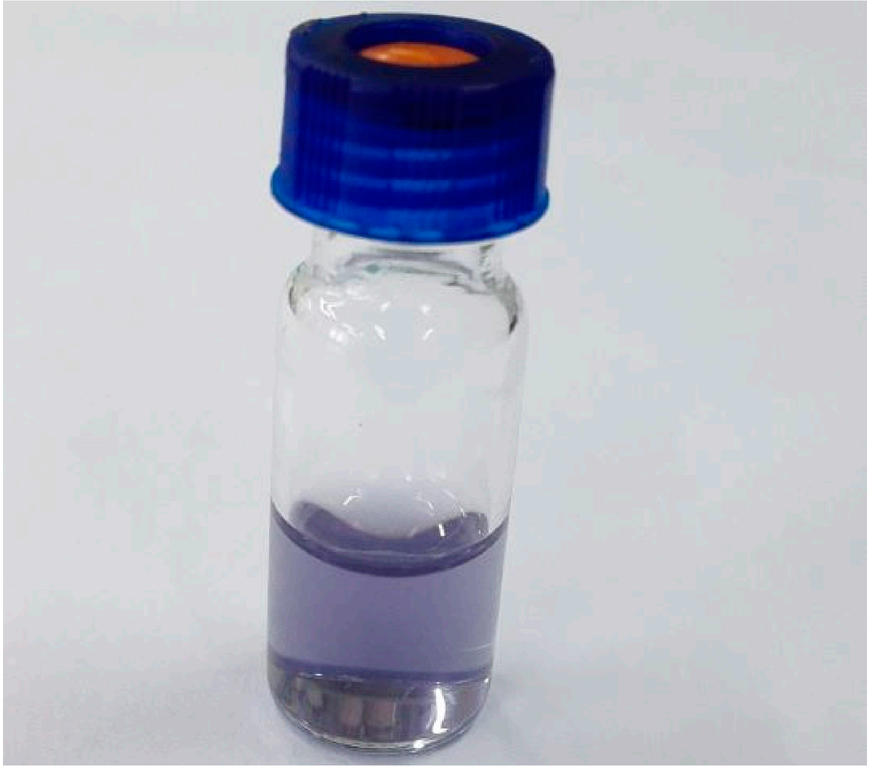

Figure S2. Appearance of DHM-Fe^{2+} complex methanolic solution.

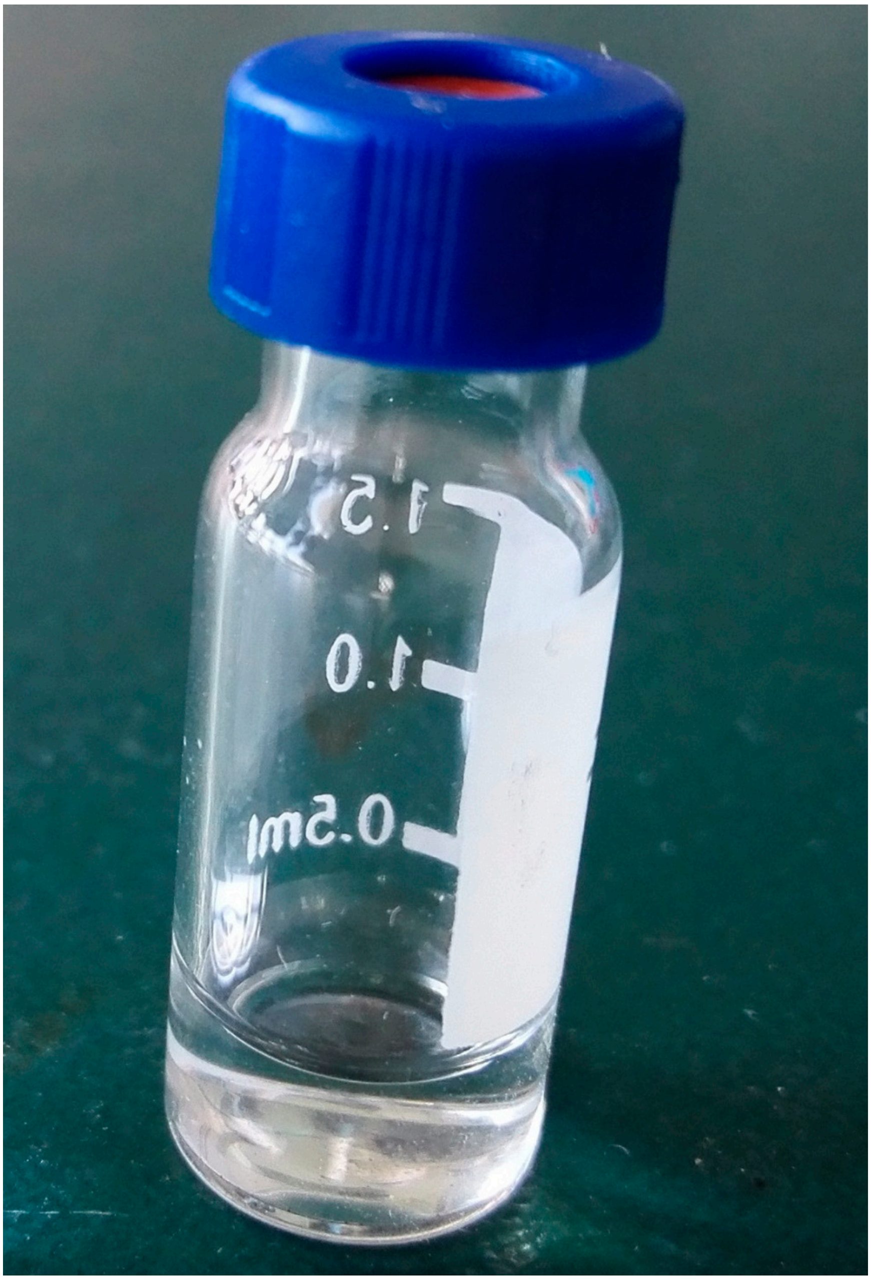

Figure S3. Appearance of DHM methanolic solution.
